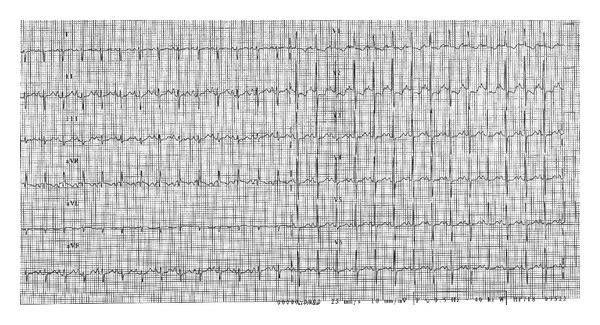# An Unusual Case of Suspected Microvascular Angina in a Newborn

**DOI:** 10.1155/2012/879161

**Published:** 2012-10-17

**Authors:** Stefania Cataldo, Giuseppe Alberto Annoni, Stefano Fiocchi, Luigi Daniele Mauri, Alessandra Corato, Gabriele Vignati

**Affiliations:** ^1^Pediatric Cardiology Unit, Ospedale Niguarda Ca' Granda, Piazza Ospedale Maggiore 3, 20162 Milan, Italy; ^2^Neonatology Unit, Ospedale Niguarda Ca' Granda, Piazza Ospedale Maggiore 3, 20162 Milan, Italy

## Abstract

Myocardial ischemia in pediatric population is uncommon and usually due to congenital heart disease or extracardiac conditions leading to poor coronary perfusion. A 6-day-old newborn presented with respiratory distress and signs of heart failure. ECG, echocardiography, and laboratory results were consistent with myocardial ischemia. Coronary angiography was performed to exclude anomalous origin of coronary arteries, showing normal coronary artery origin and course. Thrombophilia and extra-cardiac causes were ruled out. Clinical conditions improved with mechanical ventilation and diuretics, enzyme levels lowered, repolarisation and systolic function abnormalities regressed, but ischemic electrocardiographic and echocardiographic signs still presented during intense crying. Becaues of suspicion of microvascular angina, therapy with ASA and beta-blocker was started. At 5 month followup, the baby was in good clinical condition and no more episodes were recorded. We believe it is an interesting case, as no similar cases have been recorded till now.

## 1. Introduction

Myocardial ischemia is a rare condition in pediatric population. Neonatal myocardial ischemia has been reported in patients with congenital heart disease (especiallyanomalous origin of the left coronary artery from the pulmonary artery, left ventricular hypertrophy, pulmonary atresia with intact ventricular septum, truncus arteriosus, and great vessels transposition) and in extra-cardiac conditions leading to poor coronary perfusion (severe hypoxia, disseminated intravascular coagulopathy, renal artery thrombosis, and sepsis);rare causes include endocardial fibroelastosis and mediocalcinosis of the coronary arteries.In older childrenKawasaki disease and congenital heart disease (including postsurgical conditions) are the main causes, together with cardiomyopathies, substance abuse, andgenetic disorders [1, 2]. Microvascular angina is a heterogeneous entity defined as typical angina-like chest pain with electrocardiographic and metabolic evidence of myocardial ischemia but normal coronary arteries on angiography. Multiple pathophysiologic mechanisms have been described, including inflammation, estrogen deficiency, insulin resistance, enhanced pain perception due to hyperreactivity of cardiac pain receptors, and/or abnormalities in pain signal's transmission or modulation at subcortical level, but microvascular dysfunction is the most likely [3, 4]. We report a case of suspected microvascular angina in a newborn.

## 2. Case Report

**Figure 1:**
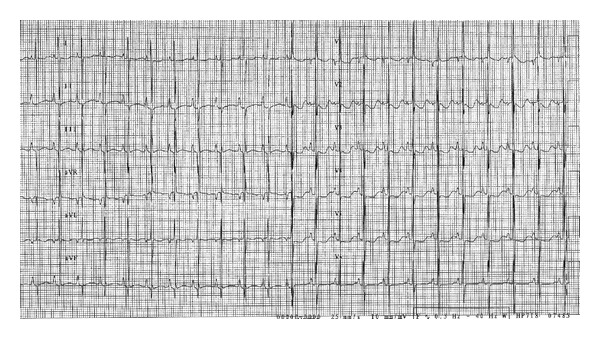


**Figure 2:**
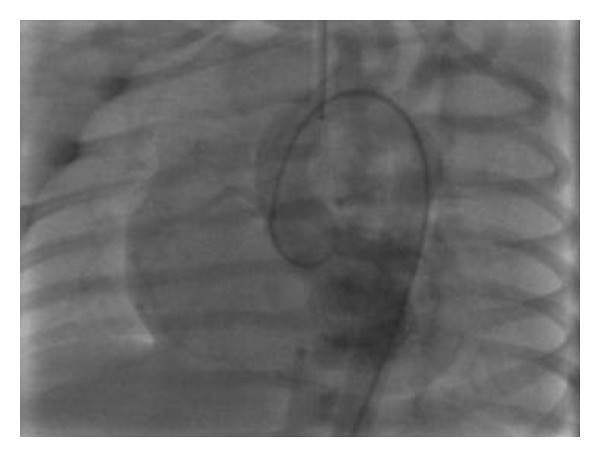


**Figure 3:**
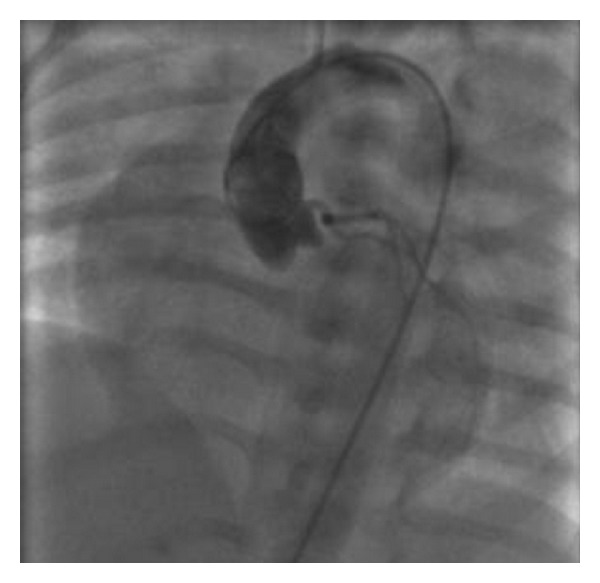


**Figure 4:**
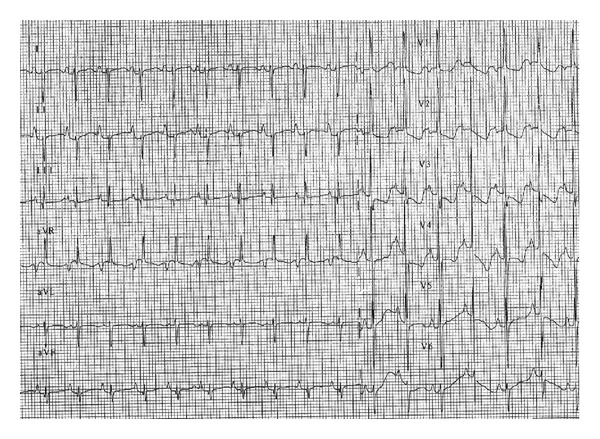


Aterm femalenewborn, weighing 3.700 kg, was born vaginally without any prenatal complications.Family history was negative for heart disease. Due to the finding of a heart murmur, transthoracic echocardiography (TTE) was performed before discharge: a moderate tricuspid regurgitation dueto valvulardysplasia was found, while all other findings were normal. The baby was dischargedin good general condition with indication for clinicalfollowup. On the 6th day of life she was admitted toERbecause oftachypnea, sweating, and failure to thrive. Body weight was 3.220 kg. Signs of heart failure and respiratory distress were present: O_2_ saturation was 96% with 6 l/min O_2_, venous blood gas analysis showed metabolic acidosis (pH 7.03, pO_2_ 46 mmHg, pCO_2_ 29 mmHg, HCO_3_—12.3 mmol/L, BE—19). Mechanical ventilation was required andthe patient was referred to our Department. Upon arrival, BP was 70/30 mmHg, HR 130 bpm, anddiuresis was preserved. Physical exam revealed a grade 3/6 systolic murmur, weakfemoral pulses, and liver was palpable 3 cm below costal arch.Laboratory studies were normal except for elevation of cardiac markers (high sensitivity troponin T 1001 ng/L, CK-MB 37.4 mcg/L) and venous blood gas analysis (pH 7.1, pO_2_ 65, pCO_2_ 28, Lac 14.8). Microbiological testswere negative for viral infection and chest radiograph showed cardiac enlargement and mild pulmonary venous congestion.ECG showed sinus rhythm133 beats/min,right atrial enlargement, inferior Q waves, and lateral repolarisation abnormalities (Figure 1). At TTEevaluation left ventricle resulted of normal size, with hypokinetic inferior wall anddyskinetic septum and mildly depressed ejection fraction and the right ventriclewas enlarged with mildly impaired systolic function. Moderate mitral regurgitation and severe tricuspid regurgitation were present. Right coronary artery (RCA) had a normal originwhileit was difficult to visualize left coronary artery (LCA). To this purpose selective coronary angiography was performed; a normal origin and anatomy of RCA and LCA were confirmed (Figures 2 and 3) and all hemodynamic parameters resulted normal, too. Left ventriculography was not performed. With mechanical ventilation and diuretic therapy hemodynamics had improved and heart failure resolved. In a few hours the baby was successfully weaned from mechanical ventilation and ECG had normalized, except for long corrected QT interval (480 msec). Cardiac necrosis markers had progressively lowered (HS TnT163.2 ng/L, CK-MB 5 mcg/L).TTE revealed normal biventricular systolic function, absence ofwall motion abnormalities, moderate tricuspid regurgitation, and no mitral regurgitation. Antithrombin III, protein C, and protein S levels were found to be normal. Ammonium, acylcarnitine panel, and plasma aminoacids levels were normal, too; urinary excretion of lactate and pyruvate was mildly elevated (40 mM/M creatinine and 59 mM/M creatinine, resp.). Diuretics were gradually discontinued. Weight increased and suction was valid, but the baby still presented sudden crisis of sweating and crying, especially during feeding or stress (i.e., diagnostic examinations, nursing care) lasting few minutes. ECG and TTE performed during one of these episodes showed a pseudonormalization of T waves followed by QRS axis rotation, lowering of ST segment in V2-V3, andsevere reduction of left ventricular ejection fraction (25%) (Figure 4). Therapy with atenolol (1 mg/kg), captopril (1 mg/kg), and aspirin (3.5 mg/kg) was started. During followup Holter ECG monitoring did not detect repolarization abnormalities and/or arrhythmias. Rest TTEafter20 days of therapyshowed left ventricle ejection fraction 50% with mild inferior wallmotion reduction and mild tricuspid regurgitation. The baby was discharged at 40 days of life, weighingkg 4.120. Polygraphic sleep monitor was prescribed. At 5-month followupthe baby wasin good general condition,weighing6 kg and there were no signs of heart failure. ECG showed normal repolarisation and corrected QT interval of 480 msec (Figure 5).

## 3. Discussion

In pediatric patients myocardial ischemia can occur as unexplained sudden death or may present with weakness, irritability, loss of consciousness,and signs of poor cardiac output. Older children can also refer chest pain, palpitation, and dyspnea. ECG manifestations do not differmuchfrom adults', but it is often not easy to differentiate between ischemic ECG and normal variants and/or ECGabnormalities associated with congenital heart disease. Electrocardiographic criteria to diagnose myocardial ischemia in children have been published (wide Q waves (>35 mm) with or without Q-wave notching, ST segment elevation (>2 mm), and prolonged corrected QT interval (>440 msec) with Q-wave abnormalities [5]), but their reliability in prospective clinical use is not known. Concerning laboratory studies, there is little evidence on the reliability ofenzymes' dosage for evaluation ofpediatric patients with suspected myocardial ischemia. Neonatal myocardial ischemia has been described in cases of abnormal coronary arteries, congenital heart disease, thromboembolic events, and perinatal asphyxia. In the case presented, a newborn without congenital heart disease developed clinical, electrocardiographic, and echocardiographic signs of subepicardial damageof the lateral and inferior myocardial wall. On the basis of clinical presentation, differential diagnosis for myocardial ischemia was entertained, including congenital heart disease and extra-cardiac causes of reduced coronary perfusion. Angiography showednormal coronaryartery origin, thus excluding the most frequent causes of ischemia in this particular population. Clinical findings were not consistent with sepsis and severe hypoxia, metabolic disorders and thrombophilia wereruled out, too. In the case presented, ischemia was induced by stress, as demonstrated by repolarisation and echocardiographic abnormalities during crying. On these bases, all findings were consistent with microvascular angina. Different therapeutic strategies have been proposed to treat adults with microvascular angina; amongantianginal drugs, beta-blockers seem to be most effective and are considered first-line treatment [6]. Even if nocasesof microvascular angina in pediatric age have been reported we decided to start beta-blocker administration in accordance to coexisting QTc prolongation. At 5 month followup no more episodes of MI were recorded. We believe that this diagnosis must be taken into account when facing clinical evidence of MI in presence of normal coronary angiography, even in small infants.It is adiagnosis per exclusionem, though, and more evidences are required to reach a better insight of this rare condition. 

## Figures and Tables

**Figure 5:**